# Gas Permeation Properties of High-Silica CHA-Type Zeolite Membrane

**DOI:** 10.3390/membranes11040249

**Published:** 2021-03-30

**Authors:** Yasuhisa Hasegawa, Chie Abe, Mayumi Natsui, Ayumi Ikeda

**Affiliations:** Research Institute of Chemical Process Technology, National Institute of Advanced Industrial Science and Technology, 4-2-1 Nigatake, Sendai 983-8551, Japan; abe-chie@aist.go.jp (C.A.); natsui-mayumi@aist.go.jp (M.N.); a-ikeda@aist.go.jp (A.I.)

**Keywords:** zeolite membrane, CHA-type zeolite, gas permeation, CO_2_ separation

## Abstract

The polycrystalline CHA-type zeolite layer with Si/Al = 18 was formed on the porous α-Al_2_O_3_ tube in this study, and the gas permeation properties were determined using single-component H_2_, CO_2_, N_2_, CH_4_, *n*-C_4_H_10_, and SF_6_ at 303–473 K. The membrane showed permeation behavior, wherein the permeance reduced with the molecular size, attributed to the effect of molecular sieving. The separation performances were also determined using the equimolar mixtures of N_2_–SF_6_, CO_2_–N_2_, and CO_2_–CH_4_. As a result, the N_2_/SF_6_ and CO_2_/CH_4_ selectivities were as high as 710 and 240, respectively. However, the CO_2_/N_2_ selectivity was only 25. These results propose that the high-silica CHA-type zeolite membrane is suitable for the separation of CO_2_ from CH_4_ by the effect of molecular sieving.

## 1. Introduction

Zeolites are microporous aluminosilicate compounds, and they have been attracted much attention as the potential material for membranes. Zeolite membranes have been studied since the 1990s, and the MFI-type zeolite membranes were formed on the substrates by deposition and intergrowth of crystallites, which were nucleated in synthesis mixtures [1,2,3,4,5,6,7,8,9,10]. Geus et al. [1] prepared MFI-type zeolite membranes on several kinds of substrates and determined gas permeation properties through the membranes. Sano et al. [2] investigated MFI-type zeolite membranes by a hydrothermal process on porous stainless-steel and alumina substrates and applied them to the separation of water/alcohol mixtures. The permeation and separation properties were also studied by several groups [11,12,13,14,15,16,17]. Moulijn and coworkers [11,12,13,14] estimated the permeation properties through the MFI-type zeolite membranes using a Maxwell–Stefan formulation containing the gas adsorption on zeolites and the diffusion inside the membrane. Morooka and coworkers [15,16] proposed an adsorption–diffusion model to discuss the effect of CO_2_ adsorption on zeolite and diffusion in the membrane for CO_2_ separation using Y-type zeolite membranes. Some kinds of zeolite membranes are available for the dehydration of many kinds of organic solvents in industries [18,19,20,21,22].

These mechanism studies have established that DDR-, CHA-, and AEI-type zeolite has favorable characteristics for the membrane material, such as small micropore diameter, large micropore volume, and the composition variability [23]. These features correspond to the permeation and separation performances and the acid and thermal stabilities of the membrane [24]. Tomita et al. developed the DDR-type zeolite membrane by the secondary growth of seeded crystals [20], and the several gas permeation properties were determined [25]. In particular, the CO_2_/CH_4_ selectivity was as high as 2000 below 250 K. However, the selectivity decreased to 220 around 300 K. Noble and coworkers investigated CHA-type silica-aluminophosphate zeolite (SAPO-34) membranes [26,27,28,29,30], and the separation performances were determined for CO_2_–CH_4_, CO_2_–N_2_, Kr–Xe, and N_2_–CH_4_ mixtures. The CO_2_/CH_4_ selectivity was 171 at 295 K [28]. Some high-silica CHA-type zeolite membranes with no phosphates were developed, and the dehydration and CO_2_ separation performances were determined [31,32]. Sato et al. developed the commercially available CHA-type zeolite membranes with Si/Al = 7, and the dehydration performances were determined for *N*-methyl-2-pyrrolidone (NMP) [31]. For a 50 wt % NMP solution at 403 K, the permeation flux and separation factor were 36 kg m^−2^ h^−1^ and 1100, respectively. Imasaka et al. developed high-silica CHA-type zeolite membranes with Si/Al = 23, and they applied them to the CO_2_ separation [32]. The membrane showed the high CO_2_ permeance (1.5 × 10^−6^ mol m^−2^ s^−1^ Pa^−1^) and CO_2_/CH_4_ selectivity (115) at 313 K. AEI-type zeolite is one of the aluminophosphate-type zeolites and contains no exchangeable cations. Since the crystal structure is similar to CHA-type zeolite, the identical CO_2_/CH_4_ selectivities were obtained [33,34,35].

Recently, we developed the rapid preparation technique for high-silica CHA-type zeolite membranes using the structure conversion of Y-type zeolite [36,37,38]. The influence of preparation conditions on the separation performances were studied, and the high reproducible procedures were determined [37]. The dehydration performances were determined for several organic solutions in our previous report [38]. However, the gas permeation and separation performances have not been determined. In this study, the gas permeation properties were determined using single-component H_2_, CO_2_, N_2_, CH_4_, *n*-C_4_H_10_, and SF_6_ at 303–473 K. The gas separation tests were also examined for binary mixtures of N_2_-SF_6_, CO_2_-N_2_, and CO_2_-CH_4_ at 303–473 K. Furthermore, the gas permeation and separation mechanisms of the CHA-type zeolite membrane were discussed in this paper.

## 2. Materials and Methods

### 2.1. Membrane Preparation

A high-silica CHA-type zeolite membrane was prepared on the outer surface of a porous α-Al_2_O_3_ support tube by the combination of the secondary growth of seed particles and the structure conversion of FAU-type zeolite [36,37,38]. The seed particles were prepared by mixing sodium hydroxide (FUJIFILM Wako, Tokyo, Japan), sodium aluminate (FUJIFILM Wako, Tokyo, Japan), a *N*,*N*,*N*-trimethyl-1-adamantammonium hydroxide solution (SDA, 25%, Sachem Asia, Osaka, Japan), and ultra-stable Y-type zeolite particles (HSZ-390HUA, Tosoh, Tokyo, Japan). The molar composition of the solution was 40 SiO_2_:1 Al_2_O_3_:4 Na_2_O:8 SDA:800 H_2_O. The mixture was poured into a Teflon-lined stainless-steel autoclave, and a hydrothermal reaction was carried out at 433 K for 4 days. Solids were recovered by filtration, washed with distilled water, and dried overnight at 383 K to obtain seed particles. For the secondary growth, a synthesis solution was prepared by the same procedures as that for the seed particles, and the mixture was stirred at room temperature for 4 h. The molar composition of the mixture was 45 SiO_2_:1 Al_2_O_3_:4.5 Na_2_O:3.4 SDA:4500 H_2_O. The α-Al_2_O_3_ tube was used as the support, and the properties were as follows: outer diameter = 2.0 mm; inside diameter = 1.5 mm; mean pore diameter = 0.3 μm; and porosity = 45%. The outer surface of the support tube was rubbed with the seed particles to implant seeds for nucleation, and the tube was added to the autoclave filled with 30 g of the synthesis solution. The autoclave was placed horizontally in an oven at 433 K for 20 h to form the polycrystalline high-silica CHA-type zeolite layer. After the autoclave was cooled to room temperature, the support tube was recovered, washed with distilled water, and dried at room temperature overnight. Finally, the tube was calcined in air at 773 K for 10 h to remove the SDA to obtain the high-silica CHA-type zeolite membrane.

The morphology was observed using a scanning electron microscope (SEM, TM-1000, Hitachi High-Technologies, Tokyo, Japan), and the composition was analyzed by an energy-dispersive X-ray (EDX) analyzer attached with the SEM. The crystal structure of the membrane was identified by X-ray diffraction (XRD, Smart-Lab, Rigaku, Tokyo, Japan).

### 2.2. Gas Permeation Test

**Figure 1:**
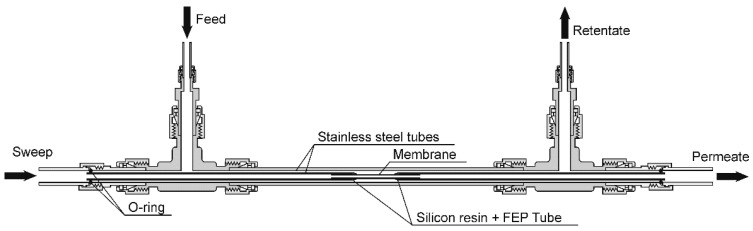
Schematic illustration of the gas permeation cell.

Both the ends of the support tube were connected to stainless-steel tubes with silicon resin (TSE3976-B, Momentive, Tokyo, Japan), and the outer surfaces of resin were wrapped with thermally shrinking tetrafluoroethylene and hexafluoropropylene copolymer (FEP) tubes (FEP-040, Junkosha, Osaka, Japan). The effective membrane area for permeation was 1.2 cm^2^. The membrane was fixed to a permeation cell, as shown in Figure 1, and the cell was placed in an electric furnace [39]. Single-component H_2_, CO_2_, N_2_, CH_4_, *n*-C_4_H_10_, and SF_6_, as well as binary mixtures of N_2_–SF_6_, CO_2_–N_2_, and CO_2_–CH_4_, were fed onto the outer surface of the membrane (feed side) at 100 mL min^−1^, and either argon (for H_2_) or helium (for the others) was introduced into the inside of the membrane (permeate side) at 10–50 mL min^−1^ as the sweep gas. The total pressures of the feed and permeate sides were kept at 300 and 101 kPa, respectively. In this study, the membrane was treated under N_2_ flow at 473 K for 30 min to remove adsorbed water, and the test gas was fed onto the feed side. The pretreatment was carried out before each measurement. The gas composition was analyzed using a gas chromatograph with a thermal conductivity detector (Shimadzu GC-8A), and the gas flow rate was determined by a soap-film flowmeter. The permeance for component *i*, *Q_i_*, was calculated using the following equation:

(1) $$Q_{i}=\frac{N_{p}y_{i}}{S(P_{fi}-P_{pi})},$$

where *N*_p_ is the molar flow rate of the outlet from the permeate side; *S*, the effective membrane area for permeation; *y_i_*, the mole fraction of component *i* in the outlet gas of the permeate side; *P*_f*i*_, the partial pressure of component *i* on the feed side; and *P*_p*i*_, the partial pressure of component *i* on the permeate side. The selectivity was defined as the ratio of the permeances in this study.

## 3. Results and discussion

### 3.1. Membrane Characterization

**Figure 2:**
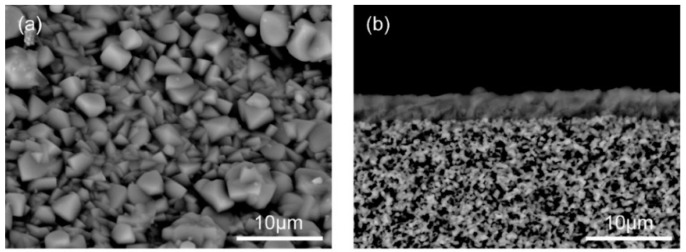
SEM images of (**a**) top surface and (**b**) fractured section of the high-silica CHA-type zeolite membrane.

**Figure 3:**
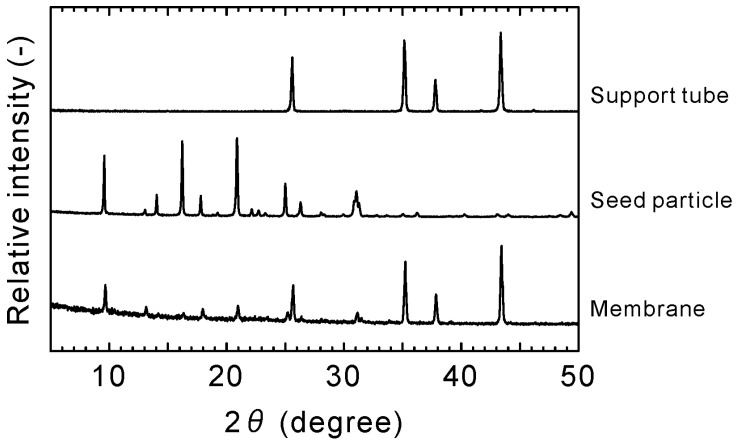
XRD patterns of the support tube, seed particles, and zeolite membrane.

Figure 2 shows the SEM images of the CHA-type zeolite membrane. The outer surface of the porous support tube was covered with a polycrystalline layer (thickness ≈ 3 μm, Si/Al = 18). The XRD pattern of the membrane contained both the peaks of the support tube and seed particles, as shown in Figure 3. These are identical to those reported previously [36,37,38]. This suggests that the polycrystalline CHA-type zeolite layer could be formed on the porous α-Al_2_O_3_ tube with high reproducibility.

### 3.2. Single-Component Gas Permeation

**Figure 4:**
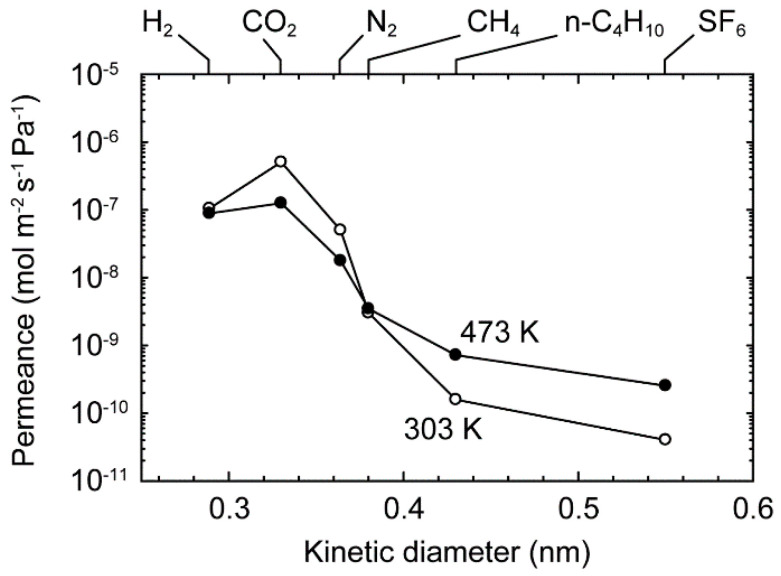
Influence of the kinetic diameters on the permeances of single-component gases at 303 and 473 K.

Figure 4 shows the influence of the kinetic diameter on the single-component gas permeance at 303 and 473 K. The permeance of CO_2_ was 5.1 × 10^−7^ mol m^−2^ s^−1^ Pa^−1^ at 303 K. The permeance, except for H_2_, decreased, with increase in the diameter, and that reached 4.1 × 10^−11^ mol m^−2^ s^−1^ Pa^−1^ for SF_6_. The diameter of the crystallographic channel aperture of the CHA-type zeolite is 0.38 nm [23], and the molecular diameters of H_2_, CO_2_, N_2_, CH_4_, *n*-C_4_H_10_, and SF_6_ are 0.289, 0.33, 0.364, 0.38, 0.43, and 0.55 nm, respectively [40]. Since H_2_, CO_2_, and N_2_ molecules are smaller than the channel diameter, those molecules can penetrate into and diffuse within the zeolite channels. Although the molecular diameter of SF_6_ is clearly larger than the channel sizes, SF_6_ was detected on the permeate side of the membrane. The marginal permeance of SF_6_ proposes that the membrane had intercrystalline boundaries. The unit cell of the high-silica CHA-type zeolite was shrunk by the air calcination, and the volume shrinkage degree was 0.6 vol % [37]. The small intercrystalline boundaries were produced by the unit cell shrinkage by the air calcination.

Moreover, the permeance of N_2_ was 1.8 × 10^−8^ mol m^−2^ s^−1^ Pa^−1^ at 473 K. After nine times heating and cooling treatment for determination of the permeation properties of single-component gases and binary mixtures, the permeance was 1.7 × 10^−8^ mol m^−2^ s^−1^ Pa^−1^. The identical permeances of N_2_ indicates that the high-silica CHA-type zeolite membrane was stable for the thermal treatment.

**Figure 5:**
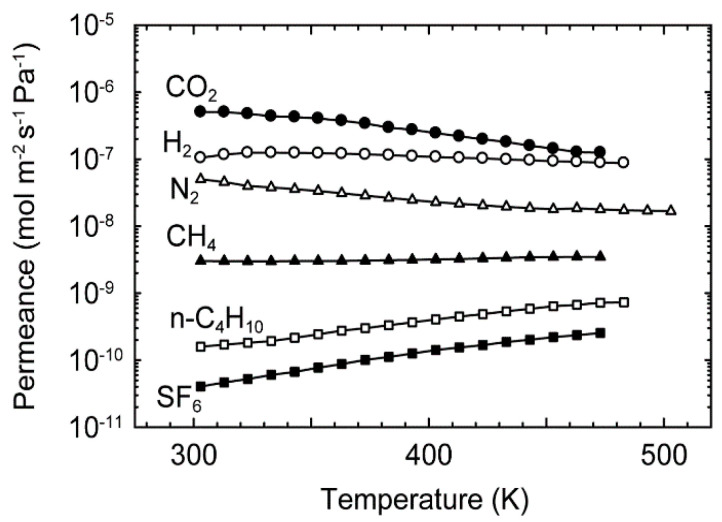
Effect of temperatures on permeances of single-component H_2_, CO_2_, N_2_, CH_4_, *n*-C_4_H_10_, and SF_6_ at 303–473 K.

Figure 5 shows the effect of temperature on the permeances of the single-component H_2_, CO_2_, N_2_, CH_4_, *n*-C_4_H_10_, and SF_6_ at 303–473 K. The permeance of CO_2_ was 5.1 × 10^−7^ mol m^−2^ s^−1^ Pa^−1^ at 303 K and decreased with temperature. As a result, it was 1.3 × 10^−7^ mol m^−2^ s^−1^ Pa^−1^ at 473 K. The permeances of H_2_ and N_2_ showed similar dependencies. Since these molecules are smaller than the channel diameter of the CHA-type zeolite, these molecules can adsorb on the zeolite channels. The adsorption amount decreased with temperature. Therefore, the permeances of H_2_, CO_2_, and N_2_ were decreased with temperature by the reduction of the concentration difference between both the sides of the membrane.

In contrast, for CH_4_, *n*-C_4_H_10_, and SF_6_, the diameters of which are identical or larger than the zeolite channels, the permeances increased with temperature. The effect of temperatures is described using the Arrhenius equation as follows:

(2) $$Q_{i}=Q_{i}^{*}\exp\left(-\frac{E_{p}}{RT}\right),$$

where Q_i_^*^ and *E*_p_ are the pre-exponential factor and activation energy for permeation, respectively. The pre-exponential factors and activation energies of single-component gas permeation are listed in Table 1. The activation energies for *n*-C_4_H_10_ and SF_6_ were higher than those of the other gases. It is well known that the difference in the diffusivities is important for the permeation through membranes [11,12,13,14,15,16,17]. The higher activation energies of *n*-C_4_H_10_ and SF_6_ suggest that it is difficult for these molecules to permeate through the intercrystalline boundaries. Therefore, the high-silica CHA-type zeolite membrane had small and minimal intercrystalline boundaries.

As shown in Table 1, the order of the activation energies was CO_2_ < N_2_ < CH_4_ < SF_6_. This suggests that the CO_2_/N_2_, CO_2_/CH_4_, and N_2_/SF_6_ selectivities are higher at lower temperatures. The separation performances for these mixtures are examined in the next subsection.

### 3.3. Binary Mixture Gas Permeation

**Figure 6:**
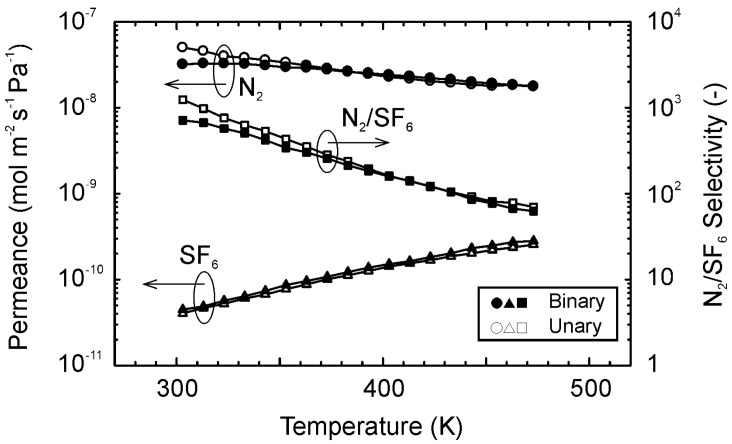
Gas permeation properties for the equimolar mixture of N_2_ and SF_6_ at 303–473 K.

Figure 6 shows the permeances of N_2_ and SF_6_ for the equimolar mixture of them at 303–473 K. As same as for the single gas, the permeance of N_2_ for the binary mixture decreased with temperature, while that of SF_6_ showed the reverse dependency. Therefore, the N_2_/SF_6_ selectivity was the highest (=710) at 303 K. The permeance of SF_6_ for the mixture was the same as that for single gas, and the permeance of N_2_ for the mixture was also identical at temperatures higher than 363 K. However, below 363 K, the permeances of N_2_ for the mixture was lower than that for the single gas. As a result, the N_2_/SF_6_ selectivity was 440 for the mixture. The lower N_2_ permeances for the mixture was attributed to the weaker interaction of N_2_ with the zeolite than SF_6_. The interaction potential of individual molecules can be described using the Lennard–Jones 12-6 equation. The depths of interaction potential are 0.59 kJ mol^−1^ and 1.85 kJ mol^−1^ for N_2_ and SF_6_, respectively [41]. The deeper potential of SF_6_ proposes that SF_6_ molecules interact with zeolites more strongly than N_2_. In addition, SF_6_ molecules permeated through the boundaries, as discussed above. Therefore, N_2_ molecules, permeated through the boundaries, were inhibited by the SF_6_ molecules, and the permeance of N_2_ became lower compared to the single gas.

**Figure 7:**
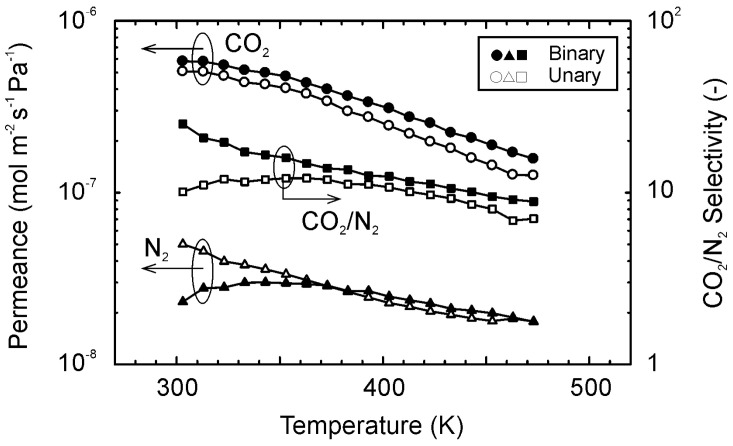
Gas permeation properties for the equimolar mixture of CO_2_ and N_2_ at 303–473 K.

Figure 7 shows the permeation properties of CO_2_ and N_2_ for the equimolar mixture of them at 303–473 K. Although the permeance of N_2_ became the maximum at 343 K for the mixture, that of CO_2_ decreased with temperature. The CO_2_/N_2_ selectivity for the mixture was the highest at 303 K (=25) and decreased with temperature. As a result, the selectivity became only 9 at 473 K.

Compared to the single gas, the temperature dependency of CO_2_ for the mixture was similar, although the permeance for the mixture was slightly higher at all temperatures. The permeance of N_2_ was almost the same that for the single gas at higher temperatures than 373 K, while it was lower below 363 K. These are typical permeation properties when molecules are transferred and separated by the preferential adsorption of CO_2_. Similar properties were also observed for the CO_2_–N_2_ separation using FAU-type zeolite membranes [15,16]. Morooka and coworkers explained the permeation properties by the preferential CO_2_ adsorption and the overtaking N_2_ by CO_2_ [15].

**Figure 8:**
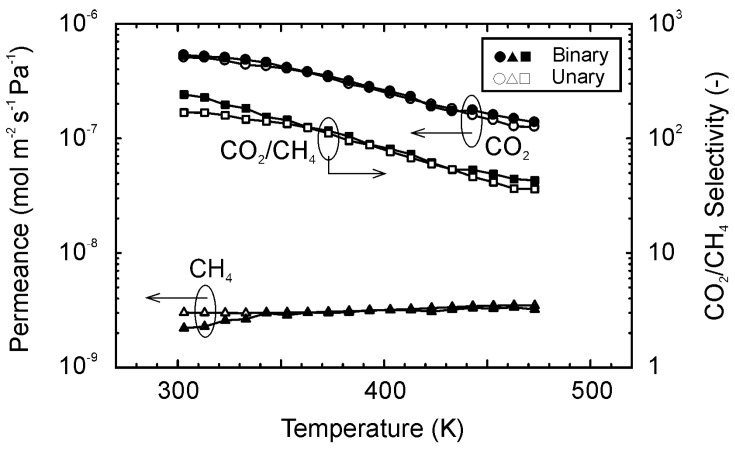
Gas permeation properties for the equimolar mixture of CO_2_ and CH_4_ at 303–473 K.

Figure 8 shows the effect of temperatures on the permeation properties of CO_2_ and CH_4_ for the equimolar mixture of them. The permeances of CO_2_ and CH_4_ for the mixture were 5.3 × 10^−7^ and 2.2 × 10^−9^ mol m^−2^ s^−1^ Pa^−1^ at 303 K, respectively. The CO_2_/CH_4_ selectivity was 240 for the mixture. The permeance of CO_2_ decreased with temperature, although that of CH_4_ showed the reverse dependency. As a result, the CO_2_/CH_4_ selectivity reduced to 43 at 473 K. Comparing to the single gases, the permeances of CO_2_ and CH_4_ for the mixture were almost identical. The permeation properties cannot be explained by only the preferential CO_2_ adsorption, as discussed in Figure 7. Since the molecular size of CH_4_ is similar to the channel diameter of the CHA-type zeolite, the diffusion of CH_4_ within the zeolite channel was slower than N_2_, as show in Figure 4. It is known that the *i*-C_4_H_10_ molecules hinder the diffusion of *n*-C_4_H_10_ for the separation of butane isomers using MFI-type zeolite membranes [39]. The CH_4_ molecules within the zeolite channels may hinder the diffusion of CO_2_ molecules.

**Figure 9:**
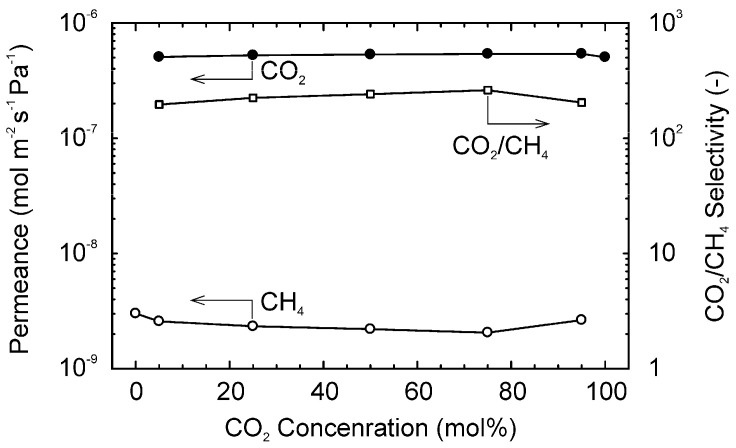
Influence of the feed gas composition on the gas permeation properties for the binary mixtures of CO_2_ and CH_4_ 303 K.

The influence of the CO_2_ concentration in the feed mixture was determined to check the interaction between CO_2_ and CH_4_ molecules during the permeation. Figure 9 shows the influences of the CO_2_ concentration in the feed mixtures on the permeation properties of CO_2_ and CH_4_ for binary mixtures of them at 303 K. If CH_4_ inhibits the permeation of CO_2_, the permeance of CO_2_ would be reduced at the dilute CO_2_ concentration. However, the permeances of CO_2_ and CH_4_ were independent of the CO_2_ concentration in the feed mixture, and the selectivity was almost constant at 200–260. The constant permeances of CO_2_ and CH_4_ propose that the CO_2_ and CH_4_ molecules did not interact each other during the membrane permeation. Therefore, it is considered that CO_2_ and CH_4_ molecules diffuse in the different passes, such as zeolite micropores and inter-crystalline boundaries.

**Figure 10:**
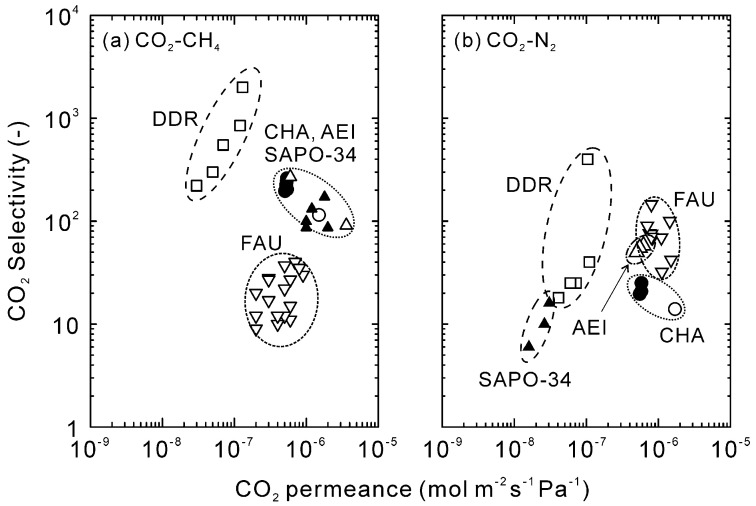
Comparison of the CO_2_ separation performance to previous reports [25,27,28,32,34,42,43].

Figure 10 compares the CO_2_ separation performance to previous reports [25,27,28,32,34,42,43]. The CO_2_ permeance and selectivity of our membrane were relatively high for CO_2_–CH_4_ mixtures, and the performances could be plotted on the trade-off line of SAPO-34, CHA-, and AEI-type zeolite membranes [27,28,32,33,34]. It is considered that the similar CO_2_ separation performances of the membranes are attributed to the similar crystal structures [44]. Although the DDR-type zeolite membrane showed extremely high CO_2_ selectivity below 300 K, the selectivity at temperature higher than 300 K was almost the same as those of CHA- and AEI-type zeolite membranes [25]. The CO_2_ selectivities of the FAU-type zeolite membranes were lower compared to those membranes [42]. On the contrary, the FAU-type zeolite membranes showed higher CO_2_ permeance and selectivity for CO_2_–N_2_ mixtures [42,43]. The selectivity of our membrane was an order of magnitude lower than those of the FAU-type zeolite membranes and comparable to those of the DDR-type zeolite membrane around 300 K. The Si/Al ratio of the DDR- and CHA-type zeolite membranes were more than 15, and the amount of the counter-cation was much less. In contrast, the FAU-type zeolite membrane contained many cations because of low Si/Al ratio (Si/Al < 2). These propose that the effect of molecular sieving such as the DDR-, CHA-, and AEI-type zeolite membranes is effective for the CO_2_ separation from CH_4_, while the selective adsorption of CO_2_ is necessary for CO_2_–N_2_ mixtures.

## 4. Conclusions

The polycrystalline CHA-type zeolite layer with Si/Al = 18 was formed on the porous α-Al_2_O_3_ tube in this study, and the gas permeation properties were determined using single-component H_2_, CO_2_, N_2_, CH_4_, *n*-C_4_H_10_, and SF_6_ at 303–473 K. The permeance was 5.1 × 10^−7^ mol m^−2^ s^−1^ Pa^−1^ for CO_2_ at 303 K and the permeance reduced with increasing the molecular size. Moreover, the permeances of H_2_, CO_2_, and N_2_ decreased with temperature, while those of CH_4_, *n*-C_4_H_10_, and SF_6_ showed reverse trends. The gas separation tests were also carried out using binary mixtures of N_2_-SF_6_, CO_2_-N_2_, and CO_2_-CH_4_. The membrane showed the high separation performance for the mixtures, and the N_2_/SF_6_, CO_2_/N_2_, and CO_2_/CH_4_ selectivities were 710, 25, and 240, respectively.

## Figures and Tables

**Table 1:** Pre-exponential factors and activation energies for single-component gas permeation through the high-silica CHA-type zeolite membrane.

Gas	*σ* (nm)	Q_i_^*^ (mol m^−2^ s^−1^ Pa^−1^)	*E*_p_ (kJ mol^−1^)
H_2_	0.289	5.5 × 10^−8^	−2.1
CO_2_	0.33	9.5 × 10^−9^	−10.6
N_2_	0.364	2.7 × 10^−9^	−7.5
CH_4_	0.38	4.7 × 10^−9^	1.2
*n*-C_4_H_10_	0.43	7.5 × 10^−9^	11.3
SF_6_	0.55	7.3 × 10^−9^	13.2

## Data Availability

Not applicable.
